# A Rapid Self-Assembly Peptide Hydrogel for Recruitment and Activation of Immune Cells

**DOI:** 10.3390/molecules27020419

**Published:** 2022-01-10

**Authors:** Ruyue Luo, Yuan Wan, Xinyi Luo, Guicen Liu, Zhaoxu Li, Jialei Chen, Di Su, Na Lu, Zhongli Luo

**Affiliations:** Molecular Medicine and Cancer Research Center, College of Basic Medical Sciences, Chongqing Medical University, Chongqing 400016, China; ruyueluo@163.com (R.L.); wanyuan@stu.cqmu.edu.cn (Y.W.); xyl@stu.cqmu.edu.cn (X.L.); Liuguicen7@163.com (G.L.); lzxzsa@stu.cqmu.edu.cn (Z.L.); cjl0917@sohu.com (J.C.); sd199877@163.com (D.S.); Lunar@stu.cqmu.edu.cn (N.L.)

**Keywords:** self-assembly peptide, hydrogel, nanofiber structures, controlled release

## Abstract

Self-assembly peptide nanotechnology has attracted much attention due to its regular and orderly structure and diverse functions. Most of the existing self-assembly peptides can form aggregates with specific structures only under specific conditions and their assembly time is relatively long. They have good biocompatibility but no immunogenicity. To optimize it, a self-assembly peptide named DRF3 was designed. It contains a hydrophilic and hydrophobic surface, using two N-terminal arginines, leucine, and two c-terminal aspartate and glutamic acid. Meanwhile, the c-terminal of the peptide was amidated, so that peptide segments were interconnected to increase diversity. Its characterization, biocompatibility, controlled release effect on antigen, immune cell recruitment ability, and antitumor properties were examined here. Congo red/aniline blue staining revealed that peptide hydrogel DRF3 could be immediately gelled in PBS. The stable β-sheet secondary structure of DRF3 was confirmed by circular dichroism spectrum and IR spectra. The observation results of cryo-scanning electron microscopy, transmission electron microscopy, and atomic force microscopy demonstrated that DRF3 formed nanotubule-like and vesicular structures in PBS, and these structures interlaced with each other to form ordered three-dimensional nanofiber structures. Meanwhile, DRF3 showed excellent biocompatibility, could sustainably and slowly release antigens, recruit dendritic cells and promote the maturation of dendritic cells (DCs) in vitro. In addition, DRF3 has a strong inhibitory effect on clear renal cell carcinoma (786-0). These results provide a reliable basis for the application of peptide hydrogels in biomedical and preclinical trials.

## 1. Introduction

Nanomaterials are materials with nanoscale (size range from 0.1 nm to 100 nm) structural units, which have been widely studied in the medical field with the development of nanotechnology [1,2]. 

Hydrogel is a material with a three-dimensional network structure [3,4], which has the characteristics of soft consistency, porosity, and high water content [5], has been widely concerned and studied in the medical field. According to the gelling mode, hydrogels can be divided into covalent hydrogels and physical hydrogels [6,7]. Its composition can be divided into a macro-molecule hydrogel and small molecule hydrogel [8,9]. 

Small molecule hydrogels are formed by the self-assembly of small molecules with molecular weight usually less than 2000 [10,11]. Molecular self-assembly is a common phenomenon in organisms, which can spontaneously assemble and form stable structures without being affected by external forces [12,13]. Biomimetic self-assembly technology of nanomaterials has become one of the main methods for the preparation of nanomaterials [14,15], which simulates the living life activities and enables nanomaterials to spontaneously form stable structures based on the interaction of non-covalent bonds. 

These self-assembly peptides are pure in composition [16], stable in performance [17], non-immunogenicity [18], non-toxic in degradation products and have good biocompatibility and has been demonstrated to be used in cell culture [19,20], drug delivery [21,22], regenerative medicine and immunotherapy [23]. At present, most peptide hydrogels are only studied as vaccine adjuvants in immunotherapy. The reason is that peptide hydrogels with good biocompatibility do not have immunogenicity, while peptide hydrogels with immunogenicity have toxic effects on cells.

Here, a self-assembling peptide, DRF3, is developed that can self-assemble into ordered nanofibers in PBS. This paper aims to prepare peptide hydrogels with good biocompatibility and antitumor properties through rational design of DRF3 molecule, natural amino acid composition, and the peptide sequence of DRF3.

## 2. Materials and Methods

### 2.1. Materials

T25/T75 breathable cell culture flask was purchased from LABSELECT (Hangzhou, China), 4% paraformaldehyde, red blood cell lysate, and 1 × PBS buffer were purchased from Biosharp (Anhui, China), 1640 medium was purchased from Gibco (Shanghai, China), and Bovine serum albumin V BSA (Albumin Bovine serum) was purchased from BioFrox (Einhausen, Germany). Cytokines IL-4 and GM-CSF were purchased from PeproteDN, 6-well, 24-well, and 96-well cell culture plates, and 96-well enzyme plates were purchased from Nest (Jiangsu, China). Trypsin was purchased from Hyclone (Logan, UT, USA). Monoclonal antibodies used in this paper were purchased from Abcam Company (Cambridgeshire, UK).

### 2.2. Synthesis of DRF3

DRF3 was designed by this research group. The sequence of DRF3 is Ac-(Arg Leu Asp Ile Lys Val Glu Phe)_2_-CONH_2_, which is a linear polypeptide synthesized from C terminal to N terminal by Chengdu CP Biochem Co., Ltd. (Chengdu, China). The molecular weight and purity of peptides were determined by mass spectrometry and HPLC.

Experimental conditions of HPLC: Mobile Phase: A: 0.1% TFA in H_2_O, B: 0.09% TFA in (80% ACN + 20% H_2_O); Flow: 1.0 mL/min 30.0%–90.0% B buffer in 20 min, 90.0% B buffer in 5 min; Column: PLRP-S 8u 300A 4.6 × 250 mm A1389#50C.

Experimental conditions of MS: Interface: ESI; Equipment: GK11010007; Nebulizing Gas Flow: 1.5 L/min; Interface Bias: +4.5 kV; CDL Temp: 250; Drying Gas Flow: 5 L/min; Block temp: 200 T; Flow: 0.2 mL/min; B.conc: 50% H_2_O/50% MeOH.

#### 2.2.1. Hydrogel Preparation

The peptide solution was prepared by dissolving 10 mg DRF3 in 1 mL sterile water. Then, 1 mL PBS was added into 1 mL peptide solution and assembled into a hydrogel at 4 °C.

#### 2.2.2. Gelling Effect

At 0, 12, 24, and 48 h after assembly, the hydrogel was stained with Congo red/aniline blue.

### 2.3. Characterization of DRF3

To observe the secondary structure of DRF3, JASCO J-815 Spectrometer (JASCO J-815, Tokyo, Japan) was used for detection.

AFM (Brooke Multimode 8, Boston, MA, USA) was used to obtain the surface topography and structure information of DRF3.

The sub-microstructure and ultrastructure of DRF3 were analyzed using low power TEM of BC200725-36: JEM-1200EX (120 KV) and high-power TEM of BC210712-86: FEI Talos F200X (FEI Corporation, Hillsboro, OR, USA).

The three-dimensional structure of DRF3 in a near-physiological state was obtained by Cryo-SEM (Hitachi SU8010, Tokyo, Japan).

The molecular structure of DRF3 was analyzed by FT-IR spectrometer (Nicolet iS10, Madison, WI, USA) and UV-3600 plus (Shimadzu, Kyodo, Japan).

### 2.4. Controlled Release

Respectively, 50 µL 2.5 mg/mL, 5.0 mg/mL and 10 mg/mL DRF3 aqueous solutions were mixed with 5 mg/mL OVA to prepare a peptide-OVA mixed solution, and incubated at 37 °C for 30 min. Then, 100 uL PBS was added and incubated at 37 °C. At hour 1, 2, 3, 5, 7, 9, 12, 24, 30, 35, 40, the upper PBS was sucked out, its absorbance at 562 nm was measured, and 100 μL PBS was added for further incubation.

### 2.5. Biocompatibility Assay of DRF3

#### 2.5.1. Cell Culture

Dendritic cells (DCs) were extracted from the healthy SPF c57 mice and cultured in RPMI-1640 medium containing 10% FBS, GM-CSF (20 ng/mL) and IL-4 (10 ng/mL) at 37 °C incubator with 5% CO_2_.

#### 2.5.2. Cell Survival Rate

PBS, LPS, OVA, DRF3, and DRF3-OVA were co-cultured with DC, respectively. PBS was negative control, LPS was a positive control. The effect of DRF3 on DC survival was analyzed by fluorescence-activated cell sorting (FACS).

#### 2.5.3. The Effect on DCs Recruitment

OVA-FITC, DRF3-FITC, and DRF3-OVA-FITC were co-cultured with DC, respectively. The effect on DCs recruitment was observed by confocal laser scanning microscope (CLSM).

#### 2.5.4. The Effect on DCs Maturation

After seven days of co-culture, a CD86 antibody was added. The effect of DRF3 on DCs maturation was observed by confocal laser scanning microscope (CLSM) and the CD86 percentage was determined by flow cytometry.

### 2.6. Cytotoxicity Assay on 786-0 Cells

#### 2.6.1. Cell Culture

The 786-0 cell line originates from humans. The 786-0 cells were purchased from BNCC and cultured in RMPI-1640 medium containing 10% FBS at 37 °C incubators with 5% CO_2_.

#### 2.6.2. CCK8 Protocol

Cytotoxicity was analyzed by cell counting kit-8 (CCK8) protocol. The cell suspension was inoculated with 1 × 10^5^ cells/mL in a 96-well cell culture plate, 100 µL per well, and incubated at 37 °C for 24 h. Then, 100 µL DRF3 with a concentration of 0.1–1 mg/mL was added, respectively and cultured at 37 °C for 24 h. 10 µL CCK8 was added to each well after a full reaction and incubated at 37 °C for 1 h. The absorbance was measured at 450 nm with a microplate reader.

#### 2.6.3. Statistical Analysis

The experimental results were replicated 3 times or more. The data were expressed by mean ± SEM, and two independent sample t-test were used to compare the experimental results between the two groups. When *p* < 0.05, the difference in experimental results was statistically significant.

## 3. Results

### 3.1. Peptide Hydrogel Morphology

#### 3.1.1. Congo Red/Aniline Blue Staining Analysis

**Figure 1:**
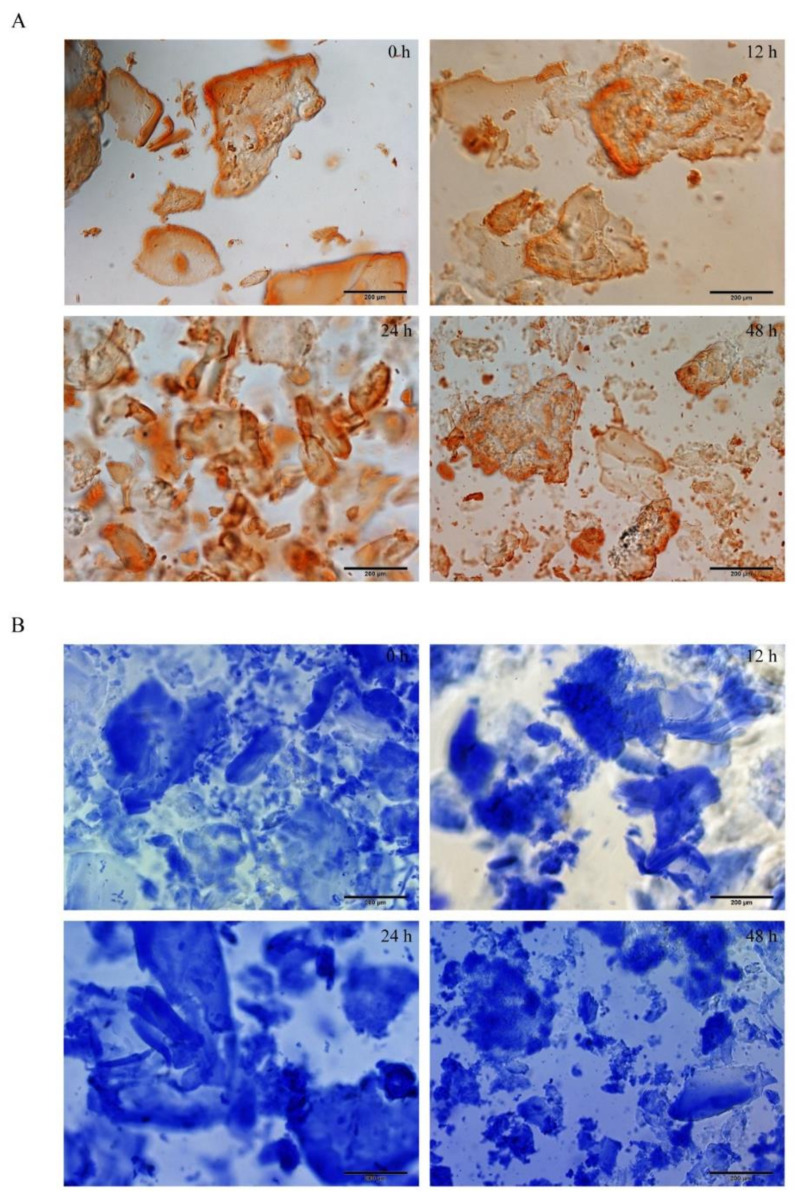
The results of the Congo red/aniline blue staining of peptide DRF3. (**A**) Congo red staining results of peptide DRF3 at 0 h, 12 h, 24 h and 48 h; (**B**) aniline blue staining results of peptide DRF3 at 0 h, 12 h, 24 h and 48 h.

Congo red/aniline blue staining showed that peptide hydrogel showed an evacuation film structure through the microscope at 0 h of self-assembling; the loose film began to assemble into a relatively dense lamellar structure at 12 h; the dense lamellar structure began to appear at 24 h and formed a dense thin film structure with stable structure and clear boundary at 48 h (Figure 1).

#### 3.1.2. Mass Spectrometry Analysis

**Figure 2:**
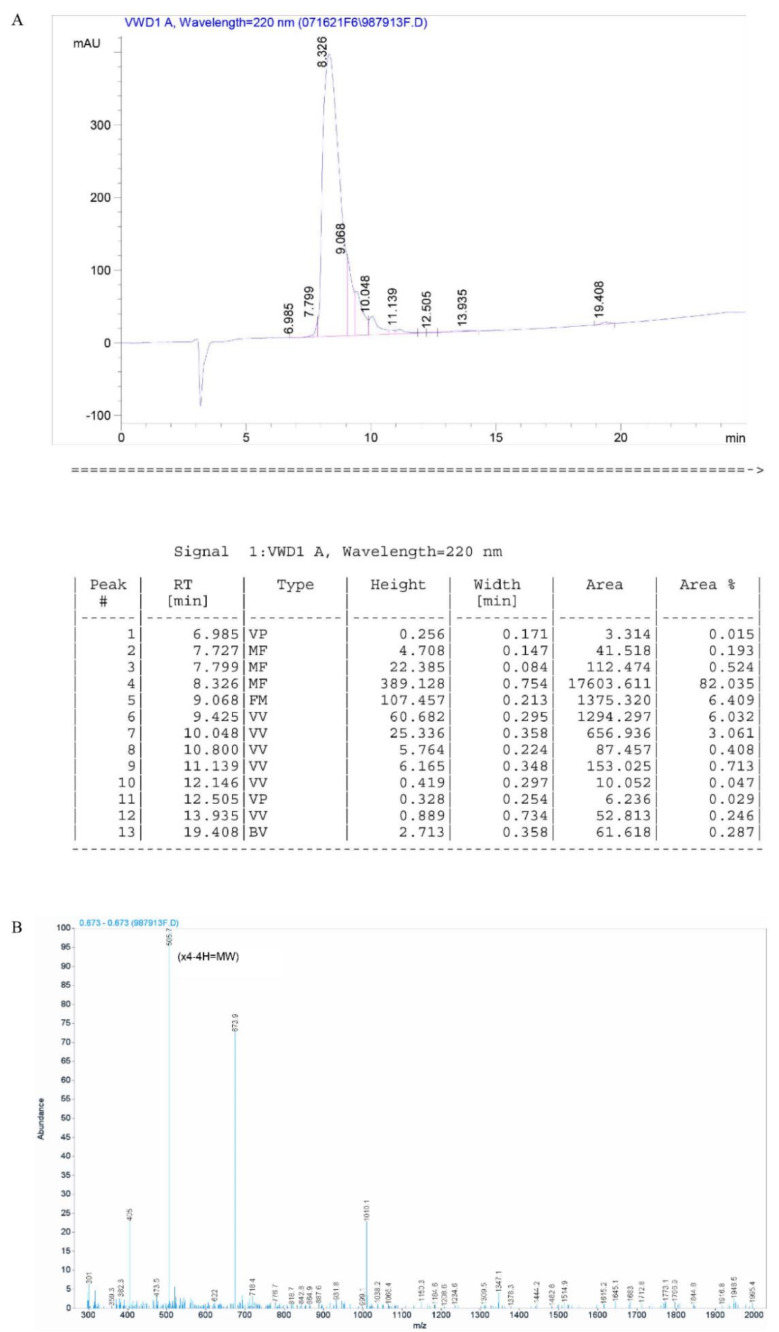
HPLC and Mass Spectrometry analysis of peptide DRF3. (**A**) HPLC analysis of peptide DRF3; (**B**) Mass Spectrometry analysis of peptide DRF3.

The relative molecular masses of DRF3 were 2019.4 (Figure 2B).

#### 3.1.3. HPLC Analysis

The purity of DRF3 was 82.3% (Figure 2A).

### 3.2. Secondary Structure of DRF3

**Figure 3:**
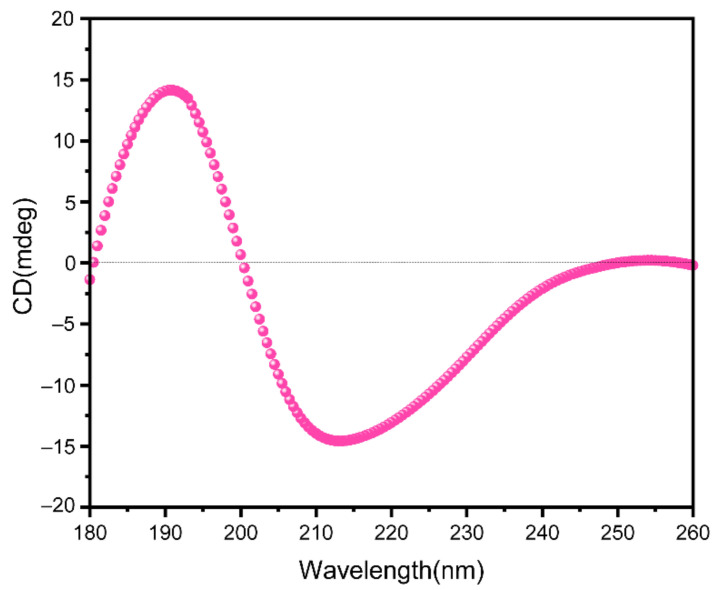
Circular dichroic analysis of DRF3.

The circular dichroism spectrum showed DRF3 has a strong positive peak at 190 nm and a negative peak at 211 nm (Figure 3).

**Figure 4:**
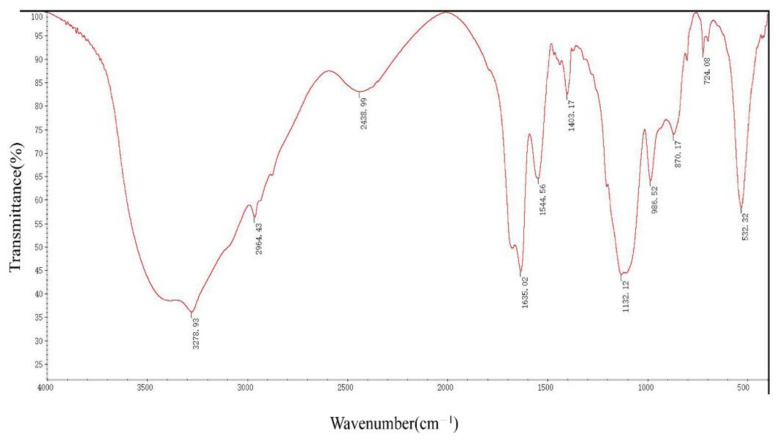
Infrared spectrogram analysis of DRF3.

DFR3′ IR peaks reveal strong absorbance peaks in the 3400–2900 cm^−1^, 1640–1600 cm^−1^, 1300–1100 cm^−1^, and 600–450 cm^−1^ areas. There are other faint peaks in the 3000–2900 cm^−1^, 2400–2000 cm^−1^, 1661–1646 cm^−1^, 1550–1450 cm^−1^, 1400–1300 cm^−1^, 1000–950 cm^−1^, 900–800 cm^−1^, and 750–650 cm^−1^ areas (Figure 4).

### 3.3. Micro-Structural Measurements of DRF3

#### 3.3.1. Cryo-SEM

**Figure 5:**
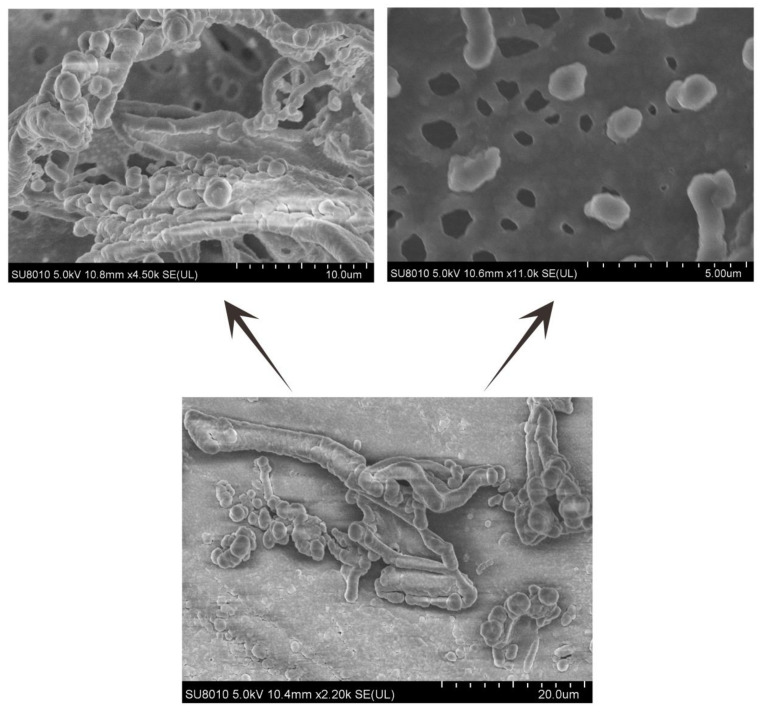
Cryogenic scanning electron microscopy (Cryo-SEM) analysis of DRF3.

DRLF3 forms nanotubule-like and vesicle-like structures in PBS under electron microscopy, which interlaced with each other to form ordered three-dimensional nanofiber structures (Figure 5).

#### 3.3.2. AFM

**Figure 6:**
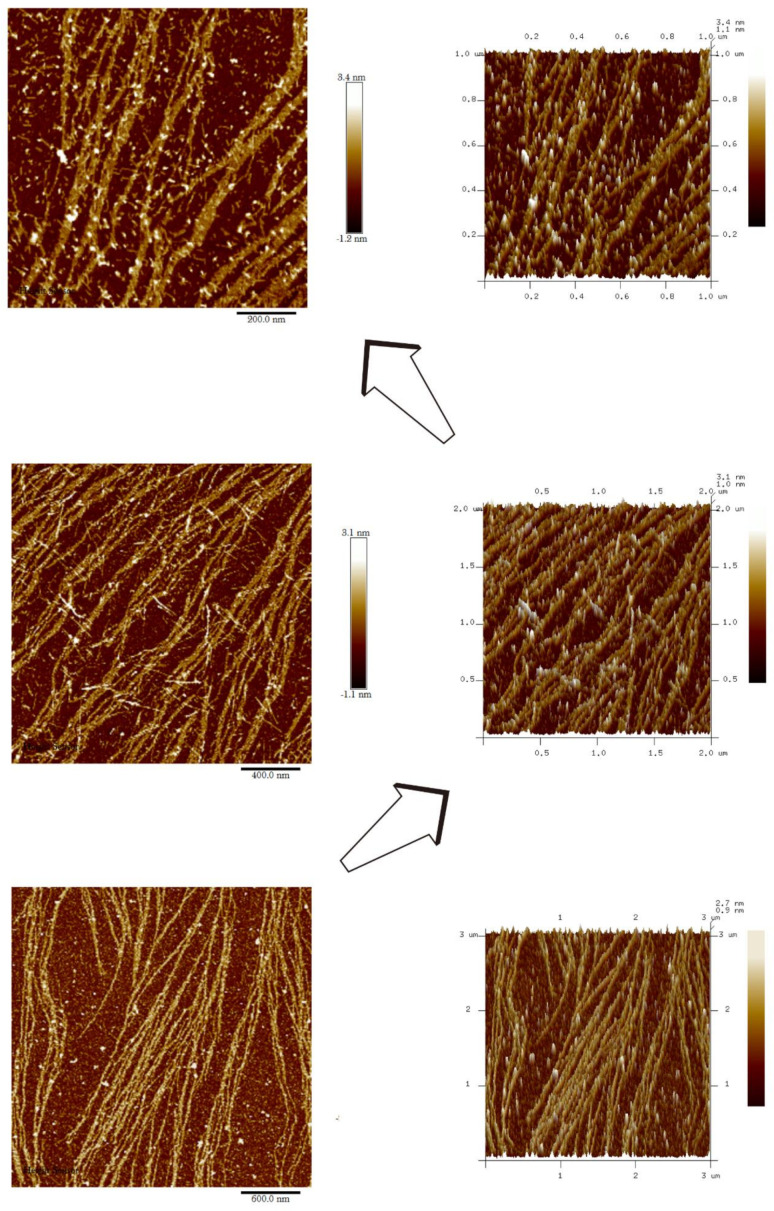
Atomic force microscopy analysis of DRF3.

AFM results reveal that DRF3 forms an ordered nanofiber structure after self-assembling. DRF3 nanofibers are about 11.6–19.2 nm in diameter (Figure 6).

#### 3.3.3. TEM

**Figure 7:**
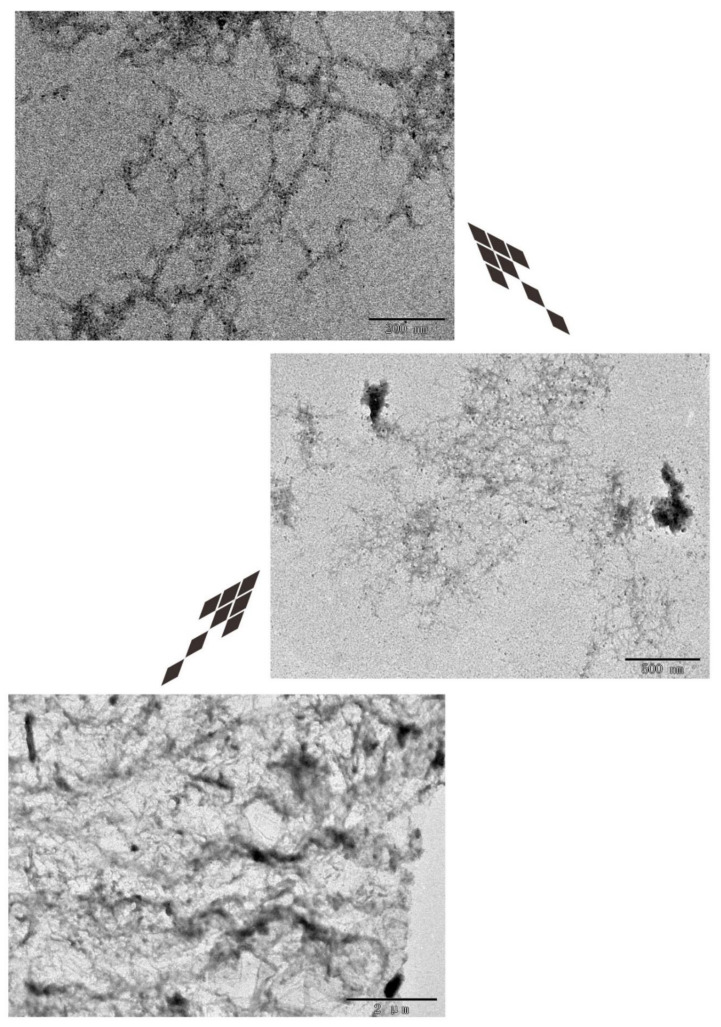
Transmission electron microscopy (TEM) analysis of DRF3.

The results of TEM are consistent with those of AFM (Figure 7).

#### 3.3.4. DRF3-FITC

**Figure 8:**
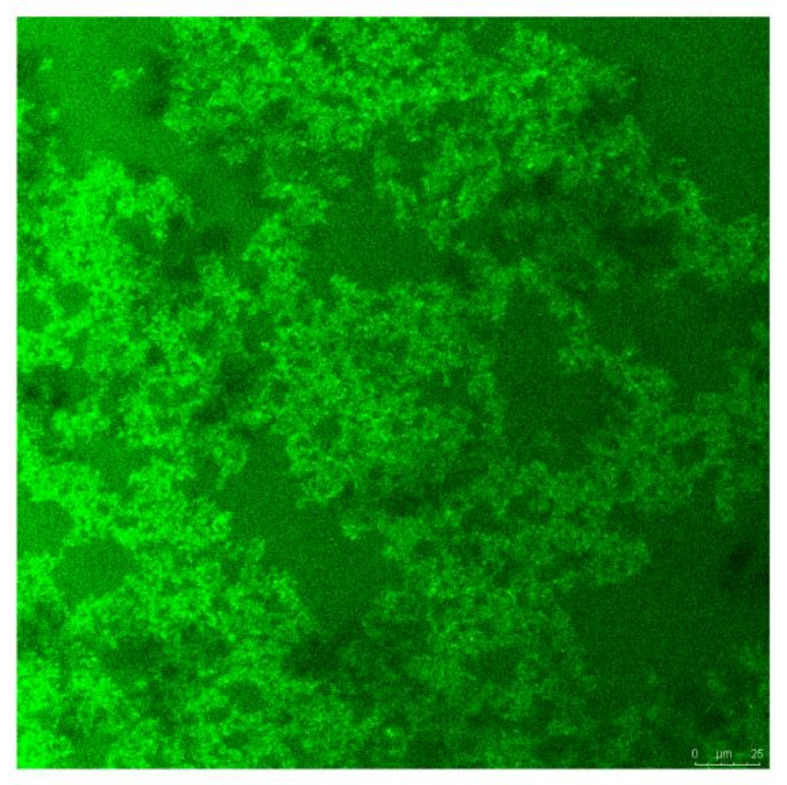
Immunofluorescence image of self-assembling peptide hydrogel DRF3-FITC in PBS.

Immunofluorescence images show that DRF3-FITC forms a dense network structure of nanofibers in PBS (Figure 8).

#### 3.3.5. Controlled Release

**Figure 9:**
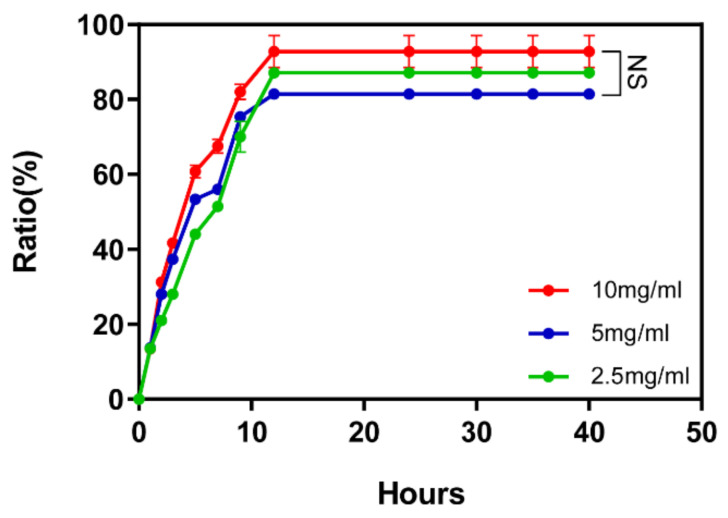
Controlled release of OVA in different concentrations of self-assembling peptide hydrogel DRF3 (The results were repeated three times, shown as mean ± SEM; NS means no significant differences *p* > 0.05).

To test the controlled release effect of DRF3, we tested its controlled release effect on OVA. The results show that at the 12th h, the three concentrations of DRF3 complete the controlled release of proteins. The controlled release rate of DRF3 to OVA reached 97% at 10 mg/mL, 82% at 5 mg/mL and 88.6% at 2.5 mg/mL (Figure 9).

### 3.4. Biocompatibility of DRF3

#### 3.4.1. Survival Rate of DCs

**Figure 10:**
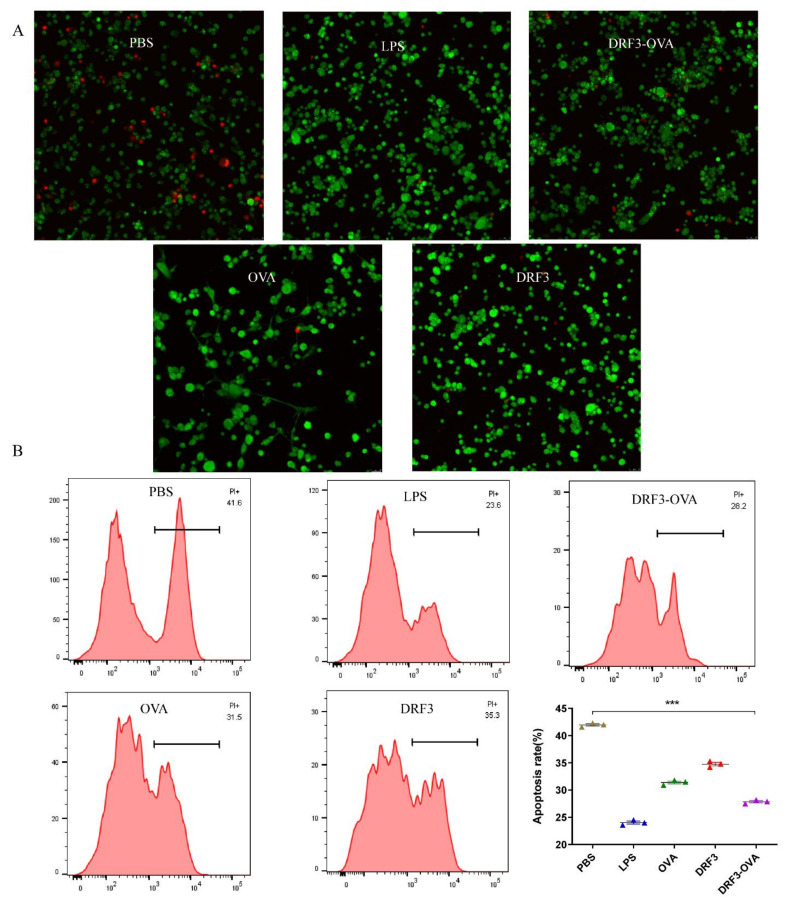
Biocompatibility detection of DRF3. (**A**) all groups were observed by confocal microscope after co-culture with DC; (**B**) the apoptosis rate of DC in each group was measured by flow cytometry (The results were repeated three times, shown as mean ± SEM; *** means significant differences *p* < 0.001).

Confocal microscope observation showed that the DC in the PBS group showed higher apoptosis, while the DC in other groups showed no obvious apoptosis (Figure 10A).

Flow cytometry was used to analyze whether the self-assembling peptide DRLF3 is toxic to DC. The apoptosis rate of DC in the PBS group was 41.6%, 23.6% in the LPS group, 31.5% in the OVA group, 35.3% in the DRF3 group, and 28.2% in the DRF3-OVA group (Figure 10B). Compared with the PBS group, the survival rate of DC in the LPS group is increased by 1.76 times, 1.32 times in the OVA group, and 1.48 times in the DRF3-OVA group. As shown in Figure 10B, the apoptosis rate of DC in LPS, OVA, DRF3, and DRF3-OVA groups is lower than that in the PBS group. Besides, the apoptosis rate of DC in the DRF3-OVA group is significantly lower than that in OVA and DRF3 group alone, indicating that DRF3 does not affect the survival rate of DC, and significantly improves the survival rate of DC when loading OVA.

#### 3.4.2. DRF3-FITC Recruits DCs In Vitro

**Figure 11:**
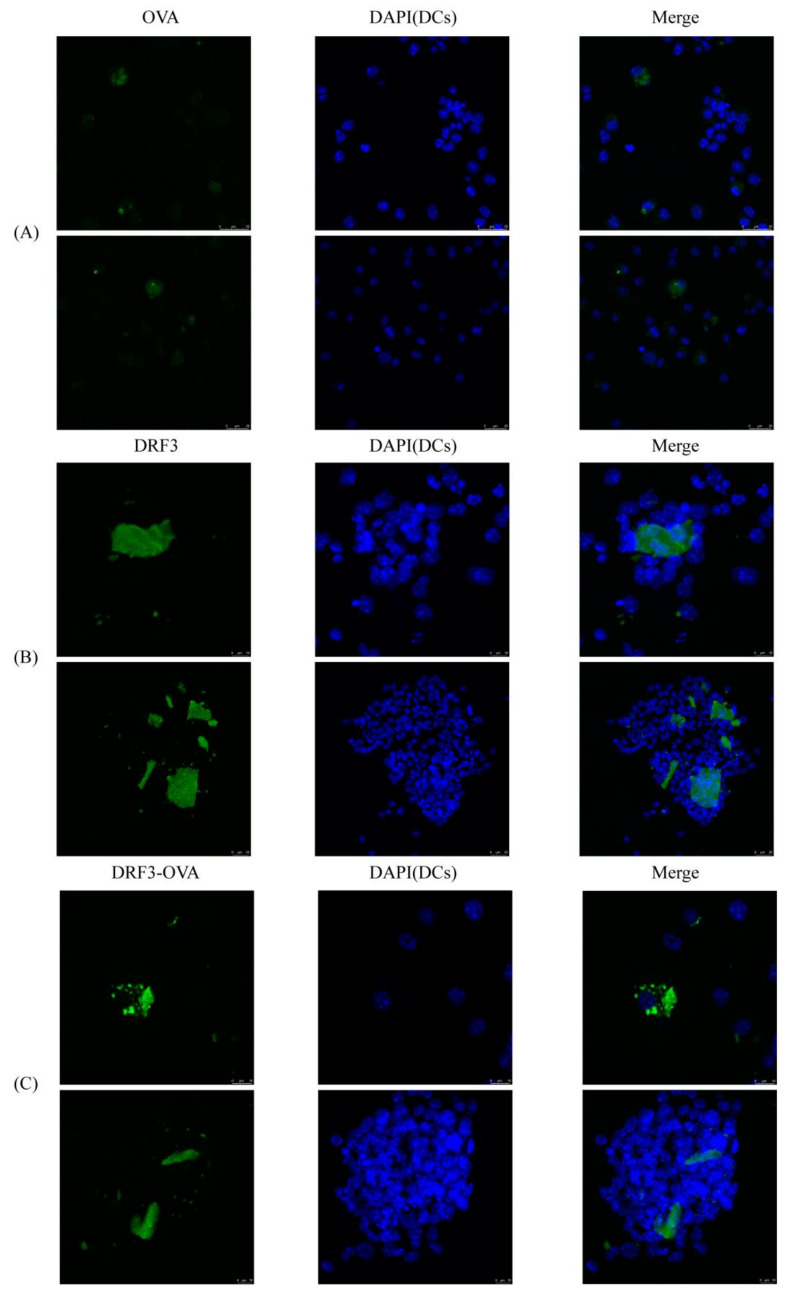
The effect on DCs recruitment. (**A**) DCs showed an aggregation growth state after OVA stimulation; (**B**) After stimulation with DRF3, DCs showed a state of largely aggregation growth, indicating that DRF3 can recruit DCs; (**C**) After being stimulated by DRF3-ova, DCs showed a large aggregation growth state, indicating that DRF3-ova can also recruit DCs, and the effect is better than DRF3 alone.

Our experimental results show that OVA-FITC, DRF3-FITC, and DRF3-OVA-FITC all stimulated immature DCs to form mature DCs. The aggregation of DCs in the OVA-FITC stimulated group was less and had no significant effect on the antigen presentation ability of DCs. DCs in the DRF3-FITC and DRF3-OVA-FITC stimulation groups showed large sheet aggregation, which preliminarily indicated that DRF3-FITC and DRF3-OVA-FITC could collect more DCs in vitro and enhance the antigen presentation ability of DCs compared with OVA-FITC (Figure 11).

#### 3.4.3. Effect on DCs Maturation

**Figure 12:**
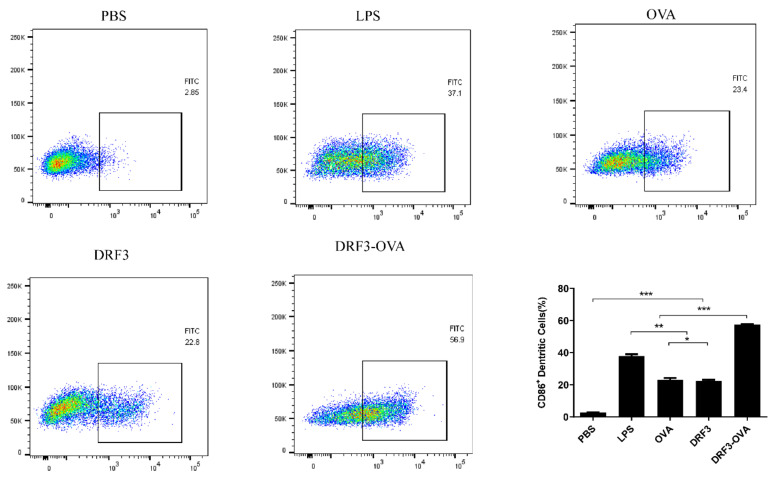
The expression rate of CD86 was detected by flow cytometry (the results were repeated three times, shown as mean ± SEM; * means significant differences *p* < 0.05; ** means significant differences *p* < 0.01; *** means significant differences *p* < 0.001).

Flow cytometry results show that the percentage of CD86 was 2.85% in the PBS group, 37.1% in the LPS group, 23.4% in the OVA group, 22.8% in the DRF3 group, and 56.9% in the DRF3-OVA group (Figure 12). Compared with the PBS group, other groups significantly increased the percentage of CD86. Meanwhile, the percentage of CD86 in the DRF3-OVA group was more than twice that in the OVA and DRF3 groups alone.

### 3.5. Antitumor Properties in Vitro

The absorbance detection of 786-0 cells stimulated by DRF3 for 24 h show that the absorbance of DRF3 is 0.376 at 0 μg/mL, 0.273 at 1000 μg/mL, 0.205 at 2500 μg/mL, and 0.119 at 5000 μg/mL. The results reveal that the tumor inhibition rate of DRF3 at 24 h is 27.4% at 1000 μg/mL, 45.5% at 2500 μg/mL and 68.4% at 5000 μg/mL.

The absorbance detection of 786-0 cells stimulated by DRF3 for 48 h show that the absorbance of DRF3 is 3.78 at 0 μg/mL, 3.32 at 1000 μg/mL, 2.97 at 2500 μg/mL, and 1.03 at 5000 μg/mL. The results demonstrate that the tumor inhibition rate of DRF3 at 24 h is 12.2% at 1000 μg/mL, 21.4% at 2500 μg/mL and 72.8% at 5000 μg/mL.

**Figure 13:**
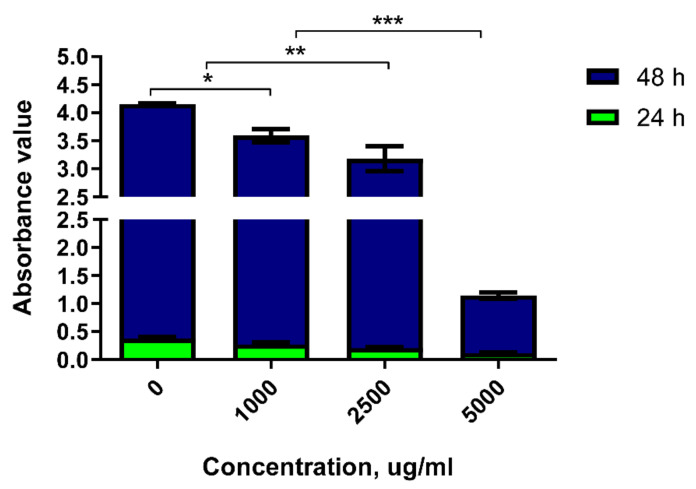
The toxicity of DRF3 on MCF-7 cells at different concentrations (the results were repeated three times, shown as mean ± SEM; * means significant differences *p* < 0.05; ** means significant differences *p* < 0.01; *** means significant differences *p* < 0.001).

The above results indicate that DRF3 has the highest tumor inhibition efficiency when the concentration is 5000 μg/mL (Figure 13).

## 4. Discussion

### 4.1. Design of DRF3

**Figure 14:**
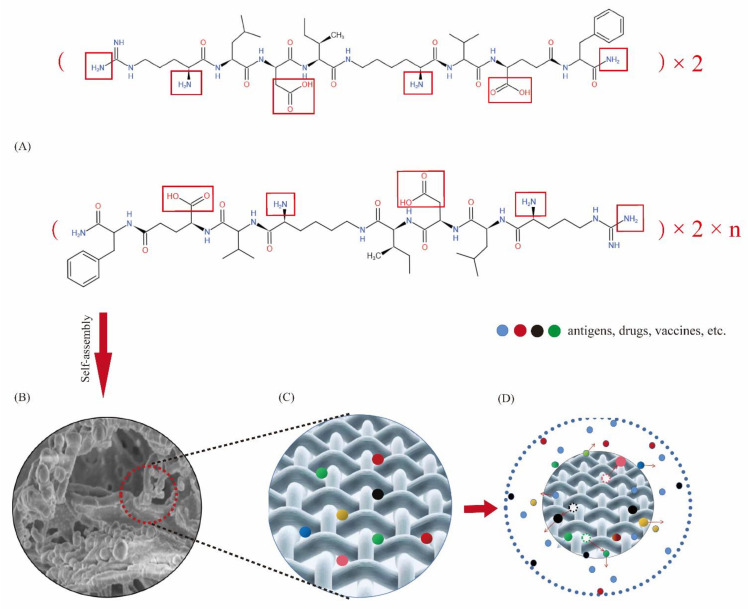
Structure diagram and reasonable model diagram of DRF3. (**A**) Structure diagram of DRF3; (**B**) Three-dimensional nanoscaffold structure model of self-assembled peptide hydrogel DRF3; (**C**) Peptide hydrogel DRF3 is loaded with antigens, drugs or vaccines; (**D**) Peptide hydrogel DRF3 controlled release antigens, drugs or vaccines, while recruiting immune cells.

Self-assembling peptide hydrogel is a biomaterial formed by using the “bottom-up” principle and existing molecular components in nature through the interaction between molecules [24,25]. Peptide molecules can spontaneously assemble into highly stable and ordered nanostructures through the interaction of non-covalent forces (hydrogen bonding [26,27], electrostatic interaction [28,29], hydrophobic interaction [30,31], van der Waals force and π-π stacking [32], etc.). DRF3 has a unique amino acid sequence (Ac-(Arg Leu Asp Ile Lys Val Glu Phe)_2_-CONH_2_), it is composed of hydrophilic amino acids and hydrophobic amino acids 1:1 alternately, among which hydrophilic amino acids are composed of alkaline amino acids and acidic amino acids 1:1 alternately to form neutral polypeptides. Besides, DRF3 uses two N-terminal arginines, leucine, and two C-terminal aspartic acid and glutamic acid, and aminated the C-terminal at the end of the peptide to increase the diversity of peptide interlinking. Furthermore, DRF3 repeats two RLDIKVEF structures, effectively increasing the length of the peptide chain and the stability of the structure, as shown in Figure 14. According to our calculation, there are a total of 24 permutations and combinations of connections. Therefore, DRF3 can be assembled instantly to form a hydrogel structure with a stable structure under the condition of neutral and normal temperature salt solution. This simple and stable assembly method lays a solid foundation for subsequent experiments, solves scientific problems such as a short peptide, long assembly time, and unstable structure (Figure 1), and also shows the potential value of DRF3 in the biomedical field.

### 4.2. Characterization of DRF3

Secondary protein units are generally considered to be stable, such as α -helix and β-sheet [33]. In the ultraviolet region of circular dichroism (190–240 nm), the main chromophore is the peptide chain [34]. The CD spectrum in this wavelength range contains information about the conformation of the main chain of biological macromolecules [35,36]. DRF3 has a strong positive peak at 190 nm, confirming that it contains an α-helix secondary structure. Meanwhile, DRF3 has a negative peak at 211 nm, indicating that it also contains β-sheet secondary structure (Figure 3). Infrared spectroscopy is one of the important methods to analyze the secondary structure of proteins. DRF3 has a strong absorption peak in the range of 1640–1600 cm^−1^ of the amide I band, revealing that DRF3 contains a β-sheet secondary structure. Besides, DRF3 has a faint absorption peak in the range of 1661–1646 cm^−1^ of the amide I band, demonstrating that DRF3 contains α-helix secondary structure (Figure 4). Accordingly, DRF3 contains both α-helix and β-sheet structures, and the content of β-sheet is more than that of α-helix. Hydrogels containing these two structures can form ordered nanofiber scaffolds through the variation of nanofibers [37]. Hydrogels are stable in vivo and vitro due to strong intramolecular forces that drive β-sheet self-assembly [38,39], which laid a foundation for subsequent related experiments of DRF3.

AFM images show that peptide hydrogel DRF3 has various sizes and forms. The nanofibers are about nm in diameter (Figure 6), which matched the TEM observation (Figure 7). The higher the hydrophobic content of peptide is, the easier it is to form the scaffold and the better its mechanical properties are [40].

Cryo-SEM images show that peptide hydrogel DRF3 can form nanotubule-like and vesicular structures in PBS (Figure 5). These structures alternate to form an ordered three-dimensional nanostructure that can be used for cell culture [41,42] and drug delivery [43].

### 4.3. Controlled Release

The results show that at the 12th hour, the three concentrations of DRF3 complete the controlled release of proteins. The controlled release rate of DRF3 to OVA reached 97% at 2.5 mg/mL, 82% at 5 mg/mL, and 88.6% at 2.5 mg/mL (Figure 9), indicating that DRF3 with different concentrations can complete the controlled release of representative antigens and ensure the sustainable effect of antigens.

### 4.4. Biocompatibility

The research demonstrates that DRF3 has good biocompatibility and was even more effective when used in combination with OVA (Figure 10). Besides, DCs in the DRF3-FITC and DRF3-OVA-FITC stimulation groups show large sheet aggregation, which preliminarily indicate that DRF3-FITC and DRF3-OVA-FITC could collect more DCs in vitro and enhance the antigen presentation ability of DCs compared with OVA-FITC (Figure 11). Furthermore, DRF3 can promote DC maturation with an increase in the positive expression rate of CD86 (Figure 12). On account of their bioadhesion, biocompatibility, and biodegradation, hydrogels have been widely used in cell culture, tissue engineering, and local delivery of various drugs/genes [44].

### 4.5. Antitumor Properties of DRF3

Ordinary hydrogels have good biocompatibility, but rarely have immunogenicity and antitumor properties [45,46]. In contrast, DRF3 has good biocompatibility and excellent antitumor properties. DRF3 has the highest tumor inhibition efficiency when the concentration is 5000 μg/mL, showing a certain degree of concentration dependence (Figure 13).

## 5. Conclusions

Peptide DRF3 with sequence Ac-(Arg Leu Asp Ile Lys Val Glu Phe)_2_-CONH_2_ was successfully synthesized, and its molecular weight and purity reached the expected standard. DRF3 is named according to its sequence. ‘D’ means ‘double’. ‘RF’ is the beginning and end of the sequence of DRF3; ‘3’ is named because DRF3 is the third peptide we synthesized. It repeats two RLDIKVEF structures, effectively increasing the length of the peptide chain and the stability of the structure. DRF3 self-assembles immediately into a colorless transparent gel and forms an ordered nanofiber structure in PBS. DRF3 has many advantages including the ability of stable, slow, and continuous controlled release antigens, good biocompatibility, recruitment of immune cells, promotion of immune cell maturation, and good antitumor ability. This research suggests that DRF3, a self-assembling peptide hydrogel, has great potential in biomedical applications and may pave the way for important medical breakthroughs.

## Data Availability

The data presented in this study are available in the article.
